# A New Method to Improve the Clinical Evaluation of Cystic Fibrosis Patients by Mucus Viscoelastic Properties

**DOI:** 10.1371/journal.pone.0082297

**Published:** 2014-01-03

**Authors:** Giovanna Tomaiuolo, Giulia Rusciano, Sergio Caserta, Antonio Carciati, Vincenzo Carnovale, Pasquale Abete, Antonio Sasso, Stefano Guido

**Affiliations:** 1 Dipartimento di Ingegneria Chimica, dei Materiali e della Produzione Industriale, Università di Napoli Federico II, Napoli, Italy; 2 CEINGE-Advanced Biotechnologies, Napoli, Italy; 3 Dipartimento di Fisica, Università di Napoli Federico II, Complesso Universitario M.S. Angelo, Napoli, Italy; 4 Dipartimento di Scienze mediche Traslazionali - Unità di Fibrosi Cistica dell’adulto, Università di Napoli Federico II, Napoli, Italy; University of Illinois at Chicago, United States of America

## Abstract

In cystic fibrosis (CF) patients airways mucus shows an increased viscoelasticity due to the concentration of high molecular weight components. Such mucus thickening eventually leads to bacterial overgrowth and prevents mucus clearance. The altered rheological behavior of mucus results in chronic lung infection and inflammation, which causes most of the cases of morbidity and mortality, although the cystic fibrosis complications affect other organs as well. Here, we present a quantitative study on the correlation between cystic fibrosis mucus viscoelasticity and patients clinical status. In particular, a new diagnostic parameter based on the correlation between CF sputum viscoelastic properties and the severity of the disease, expressed in terms of FEV1 and bacterial colonization, was developed. By using principal component analysis, we show that the types of colonization and FEV1 classes are significantly correlated to the elastic modulus, and that the latter can be used for CF severity classification with a high predictive efficiency (88%). The data presented here show that the elastic modulus of airways mucus, given the high predictive efficiency, could be used as a new clinical parameter in the prognostic evaluation of cystic fibrosis.

## Introduction

Cystic fibrosis (CF) is the most frequent life-limiting genetic disease in Caucasian populations, among whom it occurs in approximately 1 in 3000 births [1]. It is caused by mutations in a gene that encodes CF transmembrane conductance regulator (CFTR) protein which regulates the exchange of chloride and sodium ions across epithelial membranes [2-4]. The defect results in inflammation [5], infection and in thick, viscous mucoid secretions in multiple organs [6-9]. In healthy conditions, mucus is a complex fluid containing immunoglobulins, antiseptic enzymes, inorganic salts, proteins, glycoproteins known as mucins, and water. It is secreted by mucous cells and it has different functions, the most important one being acting as defense barrier against infectious agents [10,11]. From the rheological point of view, mucus is a dense, viscoelastic gel-like material, characterized by the presence of a large number of entanglements between glycoproteins and other mucosal components, stabilized by hydrogen bonding and electrostatic and hydrophobic interactions [12]. In CF patients, airways mucus, containing less water than normal, and a rather high amount of cellular debris, shows an increased viscoelasticity due to the increased concentration of high molecular weight components, especially DNA, and associated physical entanglements at the molecular level [12]. Such mucus thickening leads to abnormal mucus clearance and, finally, to bacterial overgrowth [10]. From the clinical point of view, the altered rheological behavior of mucus results in lung chronic infection and inflammation, which causes most of the cases of morbidity and mortality, despite the CF complications affect other organs as well [13]. In light of such pathological relevance, the rheological characterization of body fluids such as blood [14-17], amniotic fluid, synovial fluid [18] and mucus [10,19] have been the subject of a number of studies. Regarding mucus, two main approaches have been followed so far: i) microrheology, that is based on a magnetic microrheometer, an elegant technique for measuring rheological properties [20] of small volume of mucus [21-23], as well as multiple particle tracking [12] and dynamic light scattering [24]; the drawbacks of these techniques are related to mucus heterogeneity [12]; ii) macrorheology, where classical rotational viscometers are used [25], either under continuous and oscillatory shear [19,26]. 

In this work, we investigate the correlation between CF sputum viscoelastic properties and disease severity, in terms of FEV1% and bacterial colonization. FEV1% is defined as the ratio of the Forced Expiratory Volume in 1 second (FEV1) to the Forced Vital Capacity (FVC). The FVC is the volume of air which can be forcibly and maximally exhaled out of the lungs until no more can be expired and is usually expressed in liters, thus FEV1% indicates what percentage of the total FVC has been expelled from the lungs during the first second of forced exhalation. In the following, FEV1% is represented by just FEV1. FEV1 is typically measured by spirometry, an highly patient cooperation-dependent maneuver, that shows several disadvantages, such as reproducibility. Here, we show that CF mucus elasticity and viscosity are strictly related with bacterial colonization, and can be used as diagnostic tools in addition to (and/or replacement of) FEV1, being independent on patient cooperation. 

## Materials and Methods

### CF samples

Sputum samples were provided by the Dipartimento di Scienze Mediche Traslazionali - Unità di Fibrosi Cistica dell’adulto, following a procedure approved by the Ethics Committee of the Istituto Superiore di Sanità. Written informed consent was obtained from each individual. Sputum samples were collected in sterile containers from 33 patients (Table 1) affected by CF by voluntary expectoration during a routine clinical visit. The patients were categorized either as a function of bacterial colonization, i.e. i) Staphilococcus aureus; ii) Pseudomonas aeruginosa; iii) Burkholderia cepacia and Stenotrophomonas maltophilia, and of FEV1 value, i.e. i) FEV1 > 50% mild and moderate; ii) 30%<FEV1<50% severe; iii) FEV1 < 30% very severe. Freezing or homogenization of the sputum was not performed to avoid breakdown of structures. 

**Table 1:** Data on Age, Colonization, Sub-colonization, and FEV1 values of 33 CF donors.

# ID	Age	Colonization	Sub colonization	FEV 1 %
SA1	24	Staphiloccocus aureus	Haemophilus Influenzae	97
SA2	26	Staphiloccocus aureus	Pseudomonas aeruginosa	79
SA3	21	Staphiloccocus aureus	Haemophilus Influenzae	70
SA4	32	Staphiloccocus aureus	Haemophilus Influenzae	69
SA5	26	Staphiloccocus aureus	Haemophilus Influenzae	64
SA6	26	Staphiloccocus aureus	Haemophilus Influenzae	43
PA1	25	Pseudomonas aeruginosa	Staphiloccocus aureus	81
PA2	33	Pseudomonas aeruginosa	Staphiloccocus aureus	61
PA3	39	Pseudomonas aeruginosa	Staphiloccocus aureus	57
PA4	48	Pseudomonas aeruginosa	Staphiloccocus aureus	43
PA5	32	Pseudomonas aeruginosa	Staphiloccocus aureus	48
PA6	56	Pseudomonas aeruginosa	Haemophilus Influenzae	40
PA7	28	Pseudomonas aeruginosa	Staphiloccocus aureus	62
PA8	31	Pseudomonas aeruginosa	Staphiloccocus aureus	44
PA9	28	Pseudomonas aeruginosa	Staphiloccocus aureus	49
PA10	26	Pseudomonas aeruginosa	Staphiloccocus aureus	68
PA11	27	Pseudomonas aeruginosa	Escherichia Coli	18
PA12	28	Pseudomonas aeruginosa	Staphiloccocus aureus	34
PA13	44	Pseudomonas aeruginosa	Haemophilus Influenzae	36
PA14	23	Pseudomonas aeruginosa	Staphiloccocus aureus	32
PA15	34	Pseudomonas aeruginosa	Staphiloccocus aureus	21
PA16	34	Pseudomonas aeruginosa	Staphiloccocus aureus	59
PA17	25	Pseudomonas aeruginosa	Staphiloccocus aureus	25
PA18	27	Pseudomonas aeruginosa	Staphiloccocus aureus	68
PA19	35	Pseudomonas aeruginosa	Staphiloccocus aureus	23
PA20	57	Pseudomonas aeruginosa	Haemophilus Influenzae	40
BC1	25	Burkholderia Cepacia	Haemophilus Influenzae	40
BC2	28	Burkholderia Cepacia	Pseudomonas aeruginosa	34
BC3	40	Burkholderia Cepacia	Pseudomonas aeruginosa	25
BC4	30	Burkholderia Cepacia	Pseudomonas aeruginosa	17
SM1	24	Pseudomonas aeruginosa	Stenotrophomonas maltophilia	20
SM2	28	Pseudomonas aeruginosa	Stenotrophomonas maltophilia	23
SM3	40	Stenotrophomonas maltophilia	Pseudomonas aeruginosa	31

### Rheological measurements on CF sputum

Rheological measurements were performed using a Bohlin Instruments CVO 120 controlled-stress rheometer [27,28] operating either in continuous shear and in dynamic oscillatory mode using a 60 mm smooth stainless steel cone-plate geometry (cone angle 0.0175 rad). The sample was loaded on the plate center and possible air bubbles were removed. A steel cylindrical cage enveloping the cone-plate system has been used to prevent sample drying [29]. All the measurements were performed at room temperature to reduce sample degradation (no significant difference was found by setting the temperature to 37°C). Continuous shear tests were performed in a range of stress equal to 0.01-40 Pa, going from low stresses to high stresses and back to low values, to evaluate the possible presence of hysteresis loop. The delay time has been set equal to 4 seconds and the integration time was 20 seconds. Concerning oscillatory test, amplitude sweeps were performed to determine a stress (0.05-6 Pa) at which the response of the elastic modulus (*G*’) and loss modulus (*G*’’) was within the linear viscoelastic region of the sample. Finally, frequency sweeps (0.1-50 rad/s) were performed to determine *G’* and *G*’’ behavior. Data obtained from the rheological measurements were used to calculate useful parameters such as the magnitude of the complex modulus G* (equal to the absolute value of the sum of *G’* and *G*’’) and loss tangent, or *tan* δ (equal to the ratio between *G*’’ and *G*’) at low (1 rad/s) and high (10 rad/s) oscillation frequency. Two derivative parameters that predict mucus clearability by ciliary and cough mechanism are the Mucus Clearability index (*MCI*) and the Cough Clearability index (*CCI*) and are calculated by using the value of G* and tan δ at low and high oscillation frequency respectively, as in the following formulas [23]:

$$MCI=1.62-\left(0.22\cdot \log{G*|}_{1rad/s}\right)-\left(0.77\cdot {\tan\delta |}_{1rad/s}\right)$$

$$CCI=4.44-\left(1.07\cdot \log{G*|}_{10rad/s}\right)+\left(0.89\cdot {\tan\delta |}_{10rad/s}\right)$$

### Principal Component Analysis

Principal Component Analysis (PCA) is a statistical tool, widely used in many fields of sciences, for the analysis of multidimensional data sets [30,31]. It is used to condense the information contained in an set of eventually correlated variables (e.g. the observables associated to a physical state), into a set of uncorrelated variables called Principal Components (PCs). PCs are obtained as linear combination of the original variables. The procedure of data transformation involves the diagonalization of the correlation matrix of the initial data; as a result, the PCs are uncorrelated data, and carry the most relevant information to differentiate among the initial data. The order of the PCs denotes their importance in highlighting differences within the dataset, with PC1 describing the highest amount of variation, PC2 the second highest, and so on. The coefficients of the combination of PCs in terms of the original variables are named loadings and express the weight of each original observable to the global variance of data. The coordinates of the original data sets in the PCs space are instead referred as scores. Usually, the first 3 PCs are able to condensate more than 90% of the initial information; therefore, each set of the original data can be represented by a point in a low dimensional space (score-plot). Ideally, when plotted in the score space, points corresponding to similar data set should cluster together.

## Results and Discussion

### Rheological tests

**Figure 1:**
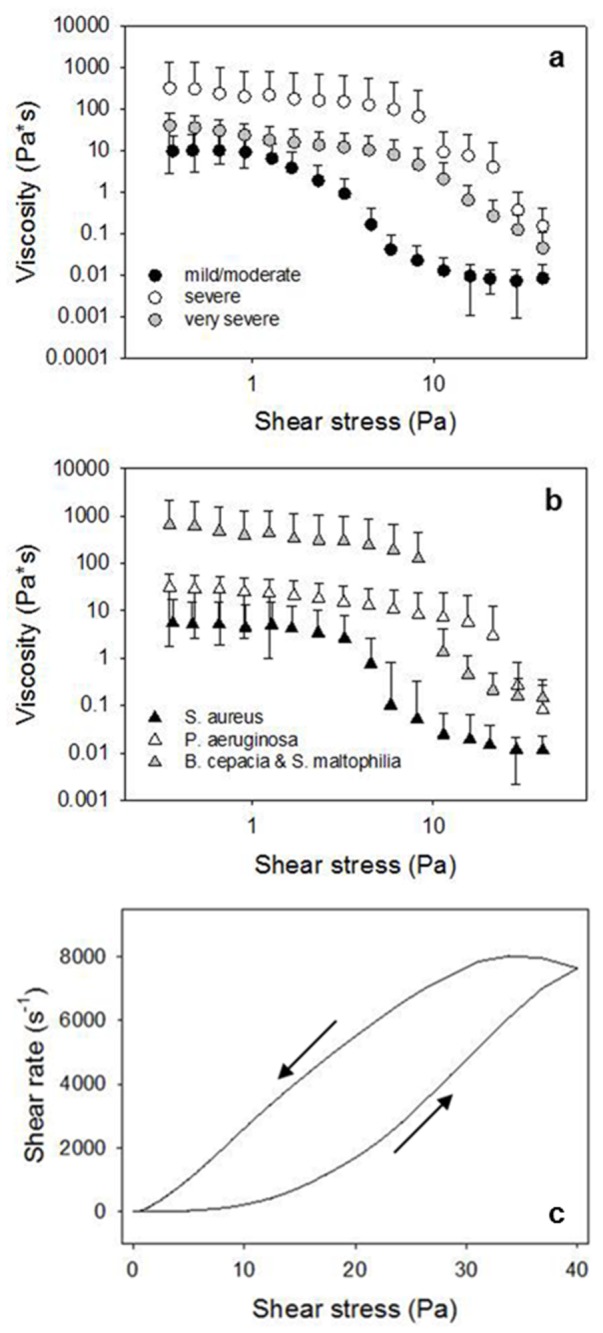
The steady state viscosity of CF sputum sample from 33 donors. a) Average viscosity from donors with different values of FEV1 (grey circle: very severe; white circle: severe; and black circle: mild/moderate) and b) Average viscosity from donors with different bacterial colonization (grey triangle: B. cepacia and S. maltophila, white triangle: P. aeruginosa and black triangle: S. aureus). Both for FEV1 and bacterial colonization, there is a significative difference between S. aureus and P. aeruginosa (P<0.05) at viscosity values below 10 Pa. c) Hysteresis loop of CF sputum.

An important property of mucus is its ability to maintain an unstirred layer of mucus adjacent to epithelial surfaces despite the vigorous shearing actions of coughing, and it does this by being a shear-thinning material that forms a lubricating plane between sliding surfaces [32]. In this work, CF sputum samples were subjected to steady state deformations of controlled shear stress to reproduce the *in vivo* conditions and study the behavior of sputum viscosity in continuous shear. The shear-thinning behavior of CF sputum is shown in Figure 1, where the average viscosity of CF sputum sample is shown as a function of the shear stress at which the sample is sheared, either for the level of severity (mild/moderate, severe and very severe depending on FEV1 values, as showed in Figure 1a) and for the type of bacterial colonization (S. aureus, P. aeruginosa, and B. cepacia and S. maltophila (Figure 1b)). As can be seen in the log-log plot, mucus viscosity shows an initial plateau zone followed by a shear-thinning region as shear stress increases: this behavior is probably due to the decreasing adhesive interaction between mucin fibers with increasing shear stress. The shear stress value at the end of the plateau (i.e., at the onset of shear thinning) is associated with a characteristic relaxation time of the material and it is different for the three cases. The classification of the CF sputum samples based on FEV1 values (Figure 1a) is not full-scale, the viscosity of very severe data (gray circle) being significantly lower than the one of severe data (white circle). In Figure 1b it is evident, instead, that classifying mucus sample using bacterial colonization, allows to distinguish the CF severity. It is known, in fact, that B. cepacia and S. matophilia lungs colonization increases the possibility of morbidity and mortality, being associated with a rapid decline in pulmonary function, more than P. aeruginosa and S. aureus [33-35]. In fact, the B. cepacia and S. maltophila set of data (grey triangle) is significantly higher than the S. aureus one (black triangle), while the P. aeruginosa set shows a halfway trend, being close to the S. aureus data at low and medium shear stress (below 10 Pa) and overcoming the B. cepacia and S. maltophila data at high shear stress. Error bars (such as in the following figures) represent the standard deviation of the measurements, which is mainly due to the quantity of saliva present in the sample that it was no possible to extract without damaging the samples. The t-test, on absolute values, was used to assess whether there is a statistically significant difference between the groups. For the mild-moderate and severe couple of data set the t-test passed for viscosities below 10 Pa both for FEV1 and bacterial colonization with P<0.05. For the severe and very severe couple of data set the t test did not pass. 

**Figure 2:**
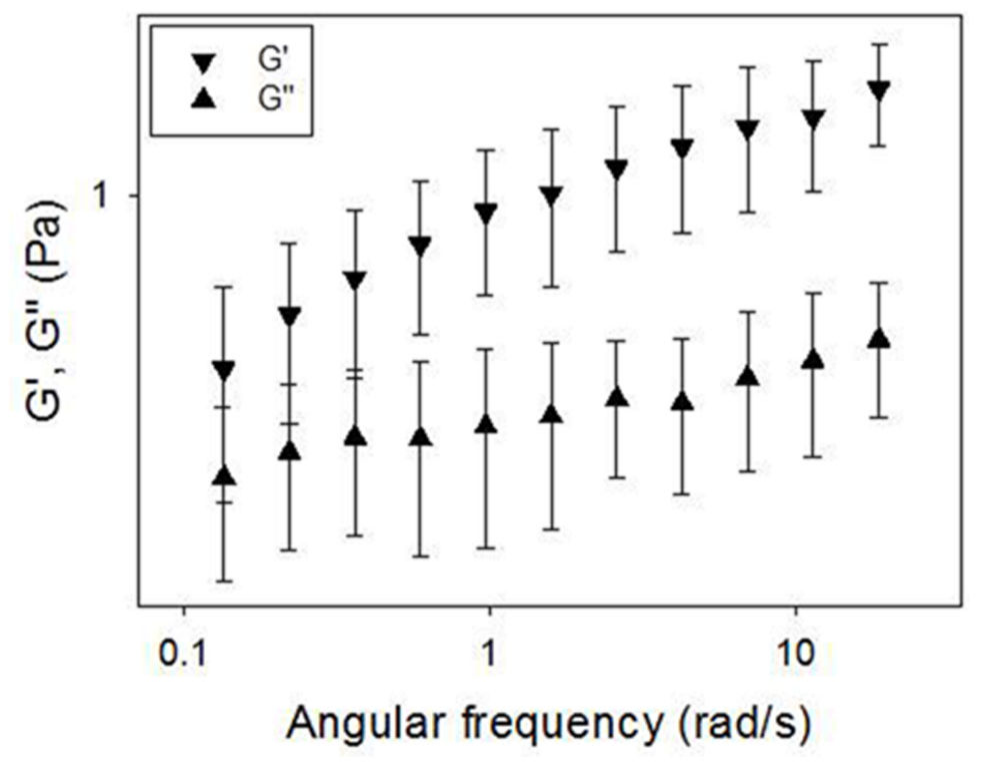
*G*’ and *G*’’ as a function of angular frequency. *G*’ and *G*’’ are almost parallel lines, typical of a cross-linked gel.

**Figure 3:**
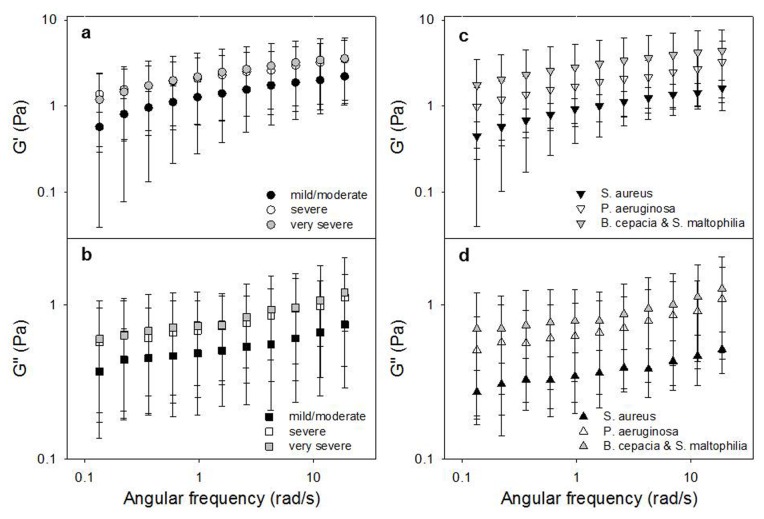
The elastic and loss modulus of CF sputum sample from 33 donors. a) *G*’ and b) *G*’’ as a function of angular frequency for the three levels of CF severity (i.e. FEV1 values corresponding to mild/moderate, severe and very severe level); c) *G*’ and d) *G*’’ as a function of angular frequency for different types of bacterial colonization (i.e. S. aureus, P. aeruginosa and B. cepacia and S. maltophila).

In Figure 1c a hysteresis loop of CF sputum is shown. This curve is obtained by increasing and subsequently decreasing the imposed shear stress. This behavior is typical of a time dependent material and it is found when an irreversible breakdown of microstructure is elicited by the action of shear flow, the material being then unable to reform its original structure in the time scale of the measurement. The first part of the up-curve (marked with an arrow going from left to right) presents an horizontal region where shear rate is around 0. The last point of this region (ca 2 Pa) corresponds to the yield value of the material, i.e. the shear stress threshold above which the material is able to flow. Below the yield point the viscosity of the material is very high. The viscoelasticity of CF sputum was characterized by applying oscillatory deformations to the samples. *G’* is the *elastic* (or *storage*) *modulus*, which is a measure of stored energy and represents the elastic component of the material *G’*’ is the *viscous* (or *loss*) *modulus*, is a measure of the energy dissipated in the material and represents the viscous component of the material. Both the elastic modulus *G*’ and the viscous modulus *G*’’ increase with frequency over a range of 0.1-50 rad/s. The elastic modulus *G*’ of CF sputum predominates over the viscous modulus *G*’’ across the entire tested range of frequency, as shown in Figure 2, where *G*’ and *G*’’ are plotted as a function of angular frequency. The *G*’ predominance as well as the very similar dependence of *G’* and *G*’’ on frequency (*G’* and *G*’’ are almost parallel lines) are typical features of a cross-linked gel. In Figures 3a and 3b, *G*’ and *G*’’ as a function of angular frequency are respectively shown for three levels of FEV1, corresponding to mild/moderate, severe and very severe. Both the elastic and viscous moduli grow with the reduction of the lungs activity and thus with the disease severity. The t-test was used to prove if there is a statistically significant difference between the groups. For the mild-moderate and severe couple of data set the t-test passed for *G*’ at low and high values of angular frequency (0.1 and 10 rad/s), with P=0.043 and 0.048 respectively, while for *G*’’ it passed only for high values of angular frequency (10 rad/s) with P=0.019, this remarking the fact that the elastic contribution is the one that mostly correlates with disease severity. Concerning the correlation with bacterial colonization, *G*’ and *G*’’ are respectively shown in Figures 3c and 3d as a function of angular frequency for S. aureus, P. aeruginosa and B. cepacia and S. maltophila. Also in this case, both the elastic and viscous moduli grow with the reduction of the lungs activity and thus with the disease severity. The t-test confirms that there is a statistically significant difference between the groups: in fact, it passed either for *G*’ and *G*’’ at each value of angular frequency. For example, for *G*’ at 1 rad/s, P=0.041 for S. aureus and P. aeruginosa data sets and at 10 rad/s P=0.008 for S. aureus and B. cepacia and S. maltophila, while for *G*’’ for S. aureus and B. cepacia and S. maltophila data sets P=0.028 and P=0.043 at 1 rad/s and 10 rad/s respectively. It is evident that the classification based on bacterial colonization works better than the one based on FEV 1. Thus, here, we propose a method to classify CF severity based on *G*’ values at 1 rad/s and on the most diffused bacterial colonization. In particular, *G*’<1.2 Pa corresponds to S. aureus, 1.2 Pa<*G*’<4 Pa corresponds to P. aeruginosa and *G*’>4 Pa corresponds to B. cepacia and S. maltophila. The advantage of this classification is that, in principle, it would be possible to identify the type of bacterial colonization in few minutes, avoiding the long time and the big cost requested by microbiological measurements. Moreover, it would be possible to isolate patient with B. cepacia, that is very aggressive in person-to-person spread. 

**Figure 4:**
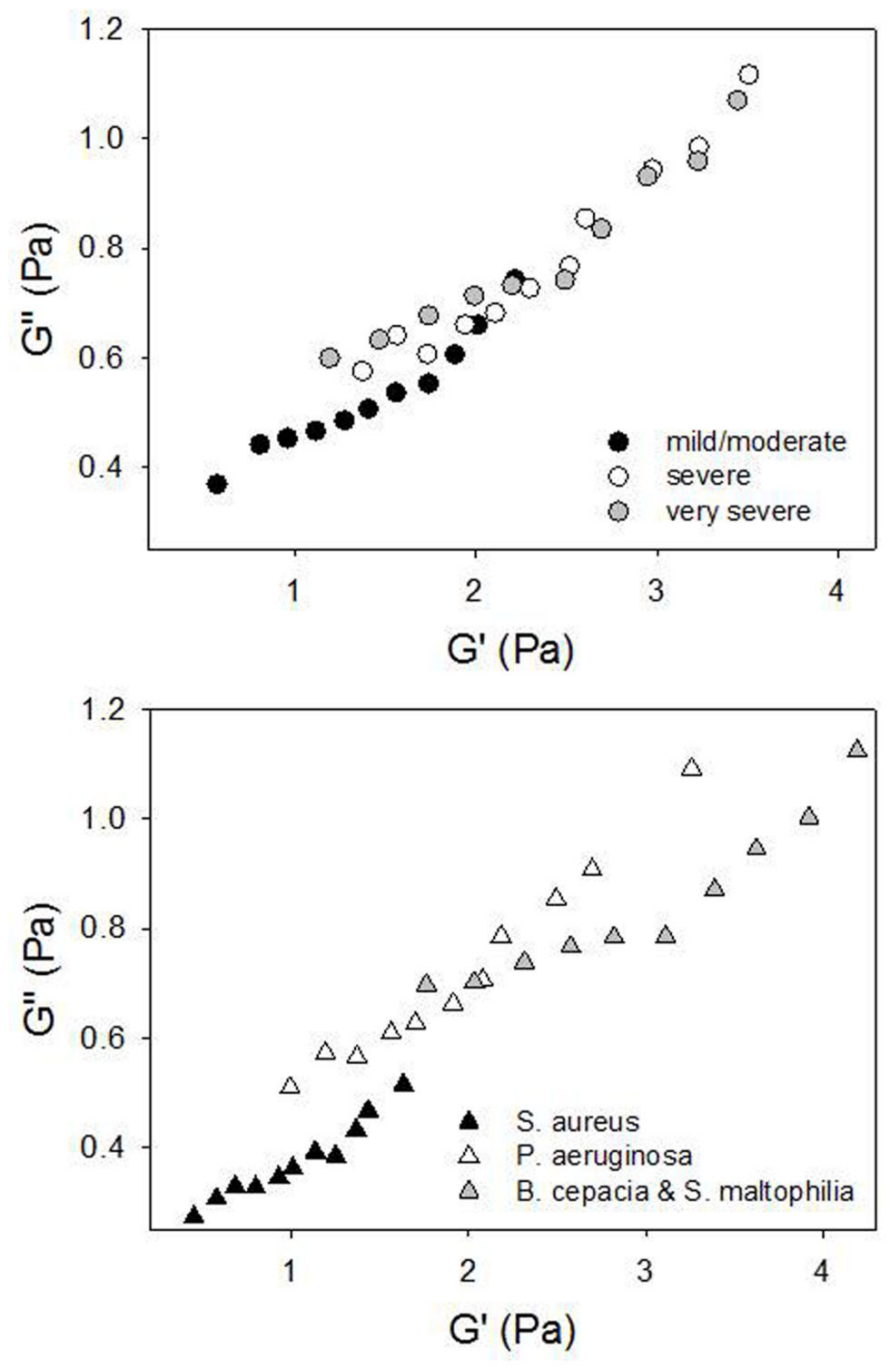
The loss modulus *G*’’(*ω*) *vs* the elastic modulus *G*’(*ω*). *ω* values range from 0.134 rad/s to 18.7 rad/s. *G*’’ vs *G*’ for a) FEV1 (grey circle: very severe; white circle: severe; black circle: mild/moderate) and b) bacterial colonization (grey triangle: B. cepacia and S. maltophila; white triangle: P. aeruginosa; black triangle: S. aureus).

In Figure 4 a plot of loss modulus *G*’’(*ω*) against storage modulus *G*’(*ω*) for the three FEV1 level of severity (Figure 4a) and for the different families of colonization (Figure 4b) is reported. As it can be seen in Figure 4a, the three sets of data classified by using the FEV1 values, nearly collapse into a single curve. This indicates that the rheological behavior of these samples, in terms of microstructure, is independent on FEV1 severity. Once again, this plot shows that the values of *G’* are higher than the ones of *G*’’ (see the scales of the axes), indicating that the elastic response of the material prevails on the viscous one. In Figure 4b, instead, the three sets of data classified by using the bacterial colonization, lie on curves of different slopes, indicating that the rheological behavior of these samples, in terms of microstructure, is dependent on colonization. For an elastic network the complex modulus and both its components depend on material parameters such as the number of elastically active entanglements and the concentration of high molecular weight species. The different slopes in Figure 4b show a different sensitivity of the moduli on such material parameters depending on disease severity. Indeed, one can distinguish three zones in the plot in Figure 4b: i) low *G*’ and *G*’’ values, where S. aureus data are found; ii) high *G’* and *G*’’ values, where there are B. cepacia and S. maltophila data and iii) intermediate values of *G’* and *G*’’, where P. aeruginosa data are found. Thus, a classification of the type of bacterial colonization can be based on this plot. 

**Figure 5:**
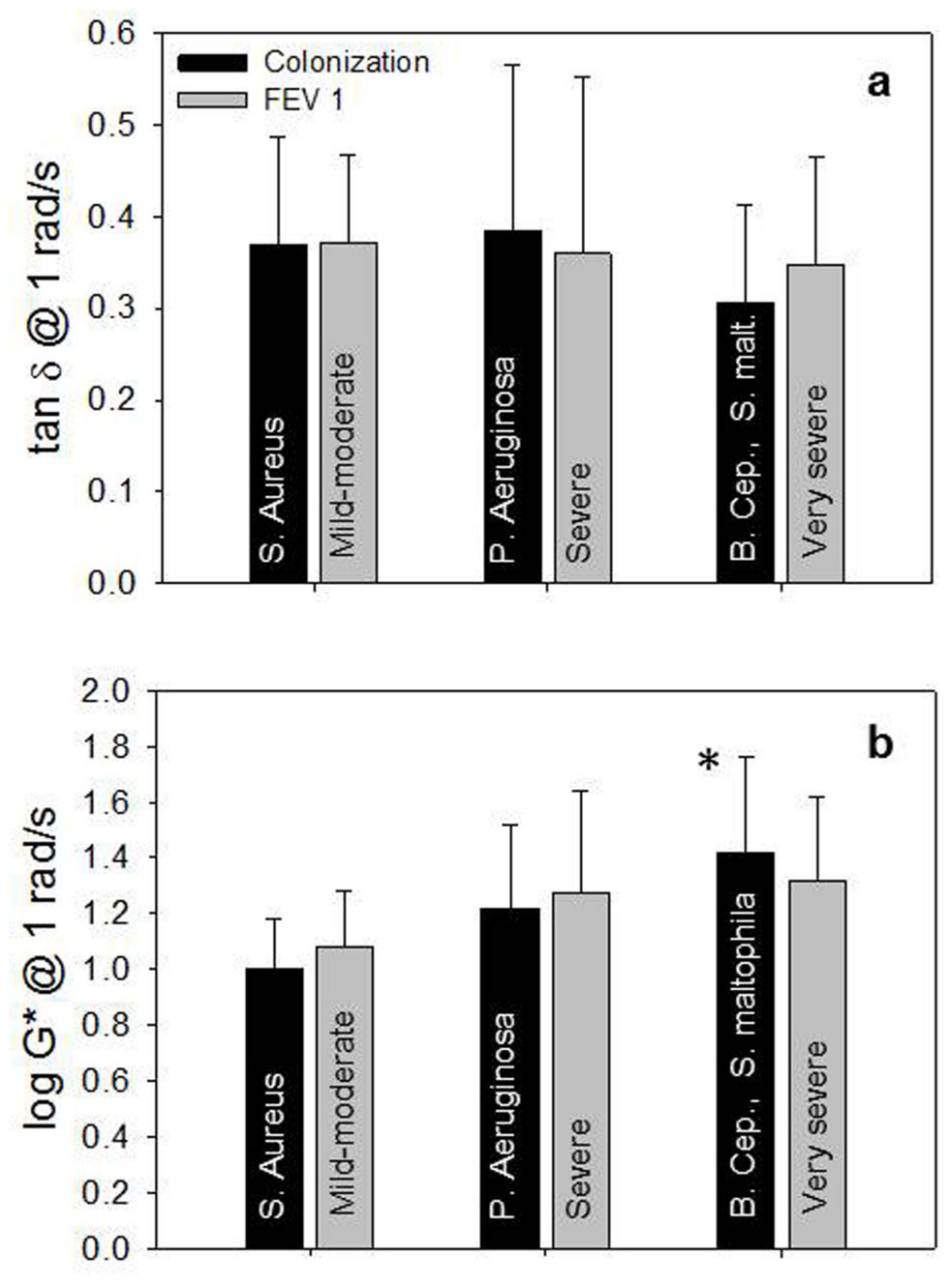
The loss tangent *tan* δ and the log value of the magnitude of the complex modulus *log* G* as a function of bacterial colonization and FEV1. a) *tan* δ (mean±SD) at 1 rad/s as a function of bacterial colonization (black) and FEV1 severity (grey). In both cases the three set of data are not significantly different one from each other; b) *log* G* (mean±SD) at 1 rad/s as a function of bacterial colonization (black) and FEV1 severity (grey). Regarding FEV1, the three set of data are not significantly different one from each other, while for bacterial colonization there is a significative difference between S. aureus and B. cepacia and S. maltophila (P=0.035).

**Figure 6:**
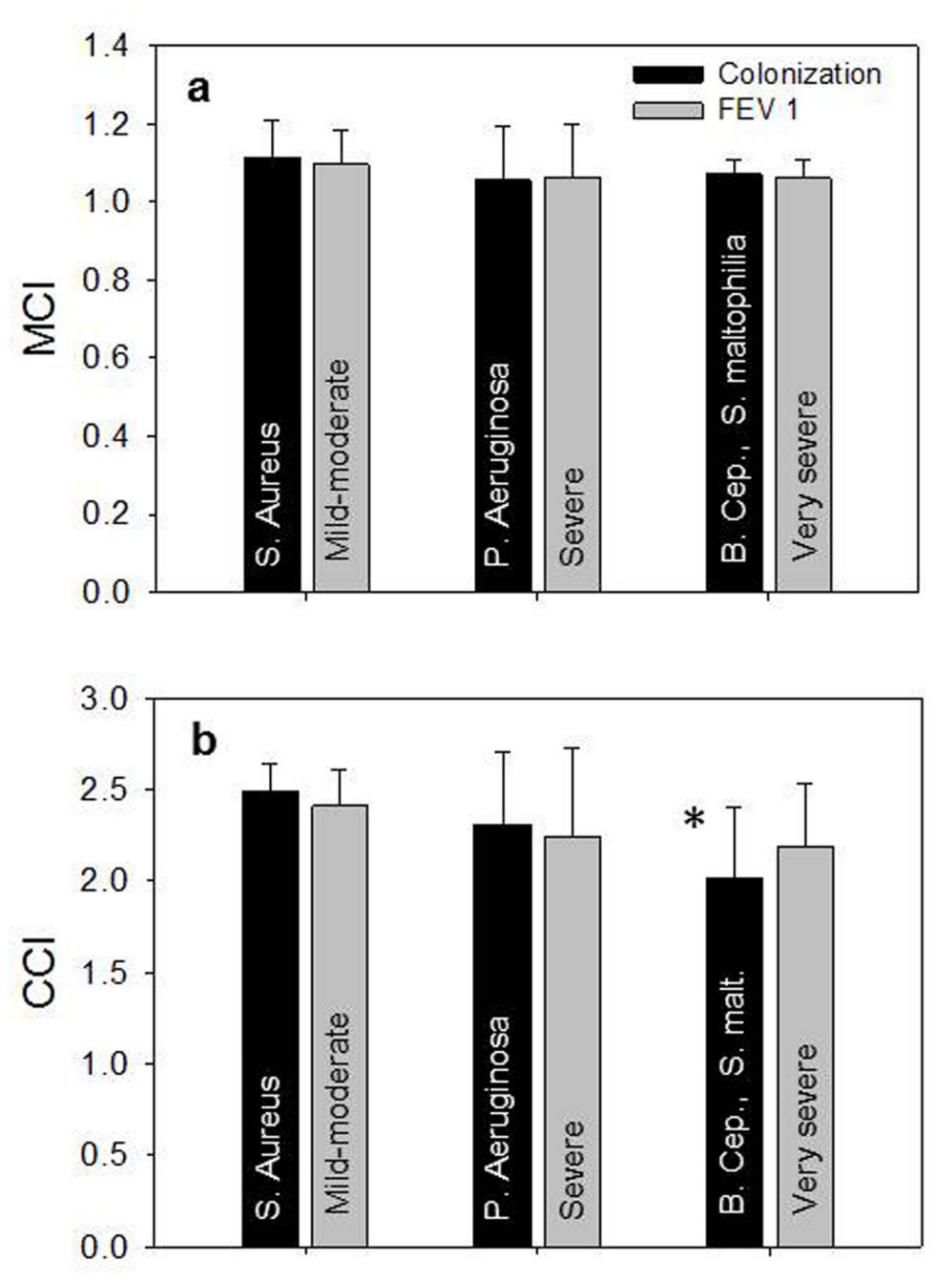
*MCI* (mucociliary clearance index) and *CCI* (cough clearance index) as a function of bacterial colonization and FEV1. a) *MCI* (mean±SD) at 1 rad/s as a function of bacterial colonization (black) and FEV1 severity (grey). In both cases the three set of data are not significantly different one from each other; b) *CCI* (mean±SD) at 10 rad/s as a function of bacterial colonization (black) and FEV1 severity (grey). Regarding FEV1, the three set of data are not significantly different one from each other, while for bacterial colonization there is a significative difference between S. aureus and B. cepacia and S. maltophila (P=0.014).

Two more viscoelastic parameters were considered, in agreement with the literature: tan δ (loss tangent) and G* (magnitude of complex modulus), each measured at 1 rad/s. The former, *tan* δ, being the ratio of *G*’’ to *G*’, represents the ratio of the viscous part to the elastic part (energy loss/energy stored) of the materials, and it is an useful quantifier of the presence and extent of elasticity in a fluid. A material that presents high (>> 1) tan δ deforms permanently when subjected to a stress and thus behaves as a liquid-like material; on the other hand, a material with low (<< 1) tan δ recoils or snaps back after the stress is removed and shows a solid-like behavior. The latter, G*, is defined by G*=*G*’+*iG*’’, where *i* is the imaginary unit. The magnitude of G* is given by $\left|G^{*}\right|=\sqrt{{\left(G'\right)}^{2}+{\left(G''\right)}^{2}}$and represents the overall resistance to deformation of a material, regardless of whether that deformation is recoverable (elastic) or non-recoverable (viscous). Here, the magnitude of G* is represented by just G*. In Figure 5a and 5b the comparison between the classification of patients by FEV1 and by colonization are respectively shown for *tan δ* and log G*: *tan δ* (Figure 5a) is almost constant with FEV1 severity, but decreases with the bacterial colonization (from S. aureus to B. cepacia and S. maltophila) indicating that lung malfunction is caused by the increased elasticity of mucus. log G*, in Figure 5b, is significantly increased in B. cepacia and S. maltophila samples as respect to S. aureus (P=0.018 and P=0.035 respectively) and this is another sign that elasticity governs mucus rheology in CF patients. For calculations of the mucociliary clearance index (*MCI*) and of the cough clearance index (*CCI*), *G*’ and *G*’’ at low frequency (0.1 rad/s) and at high frequency (10 rad/s) have been used. The low and high frequencies approximate the time scales in the airways due to ciliary beat and cough, respectively. In Figure 6a, the *MCI* shows a weak decrement with the severity of the disease either for FEV1 and bacterial colonization, while the *CCI* (Figure 6b) is significantly decreased in the case of bacterial colonization for in B. cepacia and S. maltophila samples as respect to S. aureus (P=0.014).

### Statistical analysis

**Figure 7:**
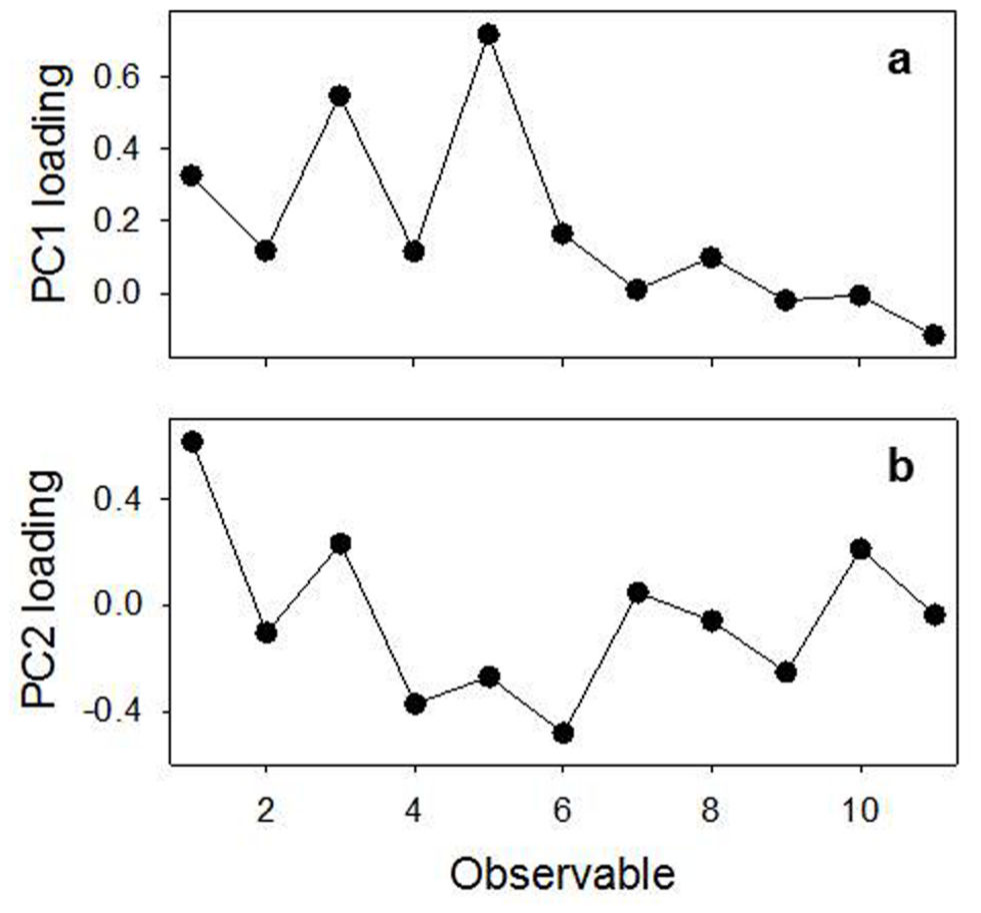
Loading plot for the first two PCs, taking globally into account 98.2% of data variability.

**Figure 8:**
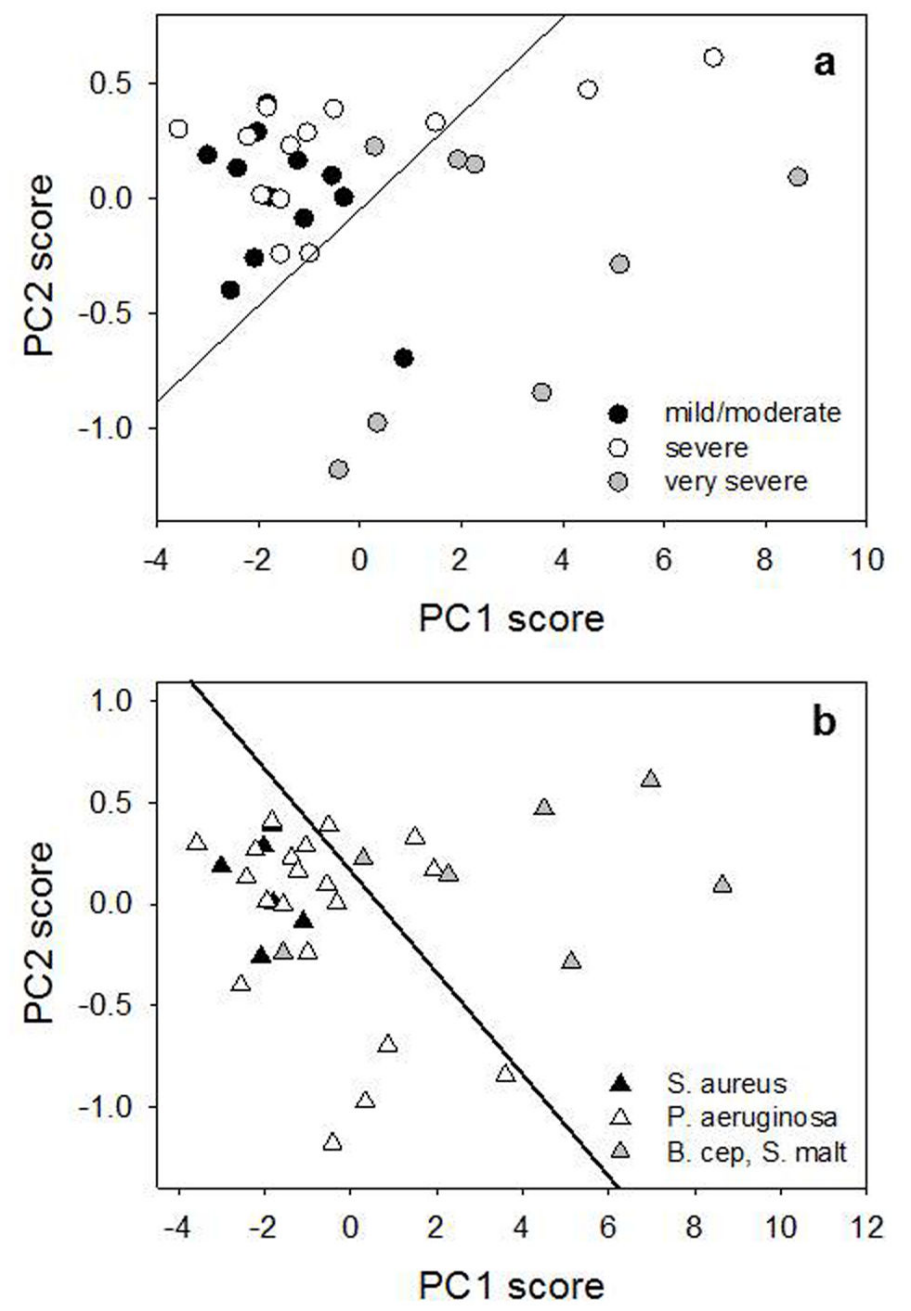
Principal Components Scatter Plot for the first two PCs. Points corresponding to the different patients are colored according to the patient bacterial colonization (a) or FEV1 class (b).

As final step, we analyzed our rheological data by PCA. This analysis was carried out in order to: *i*) test the effectiveness of our data in assessing the differences between the considered patients classes and *ii*) fully understand the relative importance of the different rheological data to classify patients. At this purpose, we associated to each of the 33 CF patients an array A_i_ of 11 selected rheological data (the so called observables), defined in Table 2. Therefore, these data were assembled in a 33x11 matrix A_ij_, with *i* running on the patients and *j* on the observables. PCA was therefore performed on this matrix by using a custom-made Matlab routine, decomposing A_ij_ in PCs. The PCA routine includes mean centering and standardization of the original raw data. Figure 7 reports the loading of the first two PCs, which globally take into account 98.2 % of the total data variability. It is interesting to note that the weight of the original rheological observables in the definition of PC1 is dominated by *G’* values (observables 1,3,5), while the contribution of *G*’’ values is strongly reduced. Moreover, the contribution of *G*’ increases with the frequency. Both results evidently confirm the experimental outcomes shown in the previous sections. Less trivial is the interpretation of PC2 loading. As for PC1, also PC2 loading shows an oscillating behavior between the *G’* and *G*’’ values; however, PC2 loading seems to suggest a contribution of MCI and CCI observables, whose values are enhanced with respect to PC1 loading. Figure 8 shows, instead, the scores of these PCs for each A_n_, colored according to FEV class (Figure 8a) or to the patient bacterial colonization (Figure 8b). As it is possible to see from this figure, PCA analysis suggests some degree of correlation of rheological data with both patients infections and FEV1 values. In the case of bacterial colonization, points relative to S. aureus- and P. aeruginosa tend clearly to clusterize in the low PC1-score region, while B. cepacia- and S. maltophila points tend to occupy the high PC1-score region. Globally, a rough divisions of patients is provided by the dashed line depicted in Figure 8b, which efficiently separates points corresponding to these two groups of patients. This suggests that there is a high correlation between the patient bacterial infection and the new rheological observables defined by PC1 and PC2. As shown in Figure 8b, points present some clusterization in the PC1-PC2 plane also according to their FEV1 class, with mild/moderate and very severe patients presenting the lowest and the highest PC1 score, respectively. Moreover, mild/moderate patients exhibit also a slight clusterization along the PC2-score coordinate, while very severe patients present a wider spread along PC2-score axis. As for bacterial colonization, it is possible to define a line separating moderate/severe patients from the very-severe one (see continuous line in Figure 8b). Interestingly, two of the four apparently misclassified patients (BC2 and SM3) presents a FEV1 value which is border-line between the severe and very-severe FEV class (34 and 31, respectively). To test the effective reliability of PCA in discriminating CF patients according to both their bacterial infection and/or FEV1 class, we used the leave-one-out cross validation procedure (LOOCV) [36]. 

**Table 2:** Rheological observables used for PCA.

1	2	3	4	5	6	7	8	9	10	11
G’0.1	G’’	G’	G’’	G’	G’’	η	Log G*	tan δ	MCI	CCI
rad/s	0.1 rad/s	1 rad/s	1 rad/s	10 rad/s	10 rad/s	10 Pa	1 rad/s	1 rad/s		

More specifically, the $\bar{j}$ ^th^ column of this table defines the observable of the column $\bar{j}$ of the matrix of data A_ij_ (j=$\bar{j}$, i running on the [1-33] interval).

The results of this analysis were used to calculate the so called ‘confusion matrix’, in which all the correct guesses are located on the diagonal of the matrix (true positive, TP and true negative, TN), while misclassified data (false positive, FP and false negative, FN) are represented by the off-diagonal elements. From this matrix, the accuracy A=(TP +TN)/(TP+TN+FP+FN) was calculated.

The confusion matrices for the two cases analyzed herein are reported below (Table 3 and 4). From their analysis it is possible to estimate a classification efficiency of about 88% in both cases. This is a quite good result, considering the rather limited number of patients analyzed in this study, and it holds promise for the development of an high efficient algorithm for CF patients classification only based on the measurement of objective rheological parameters.

**Table 3:** Confusion matrix for CF-patients, classified according to their bacterial colonization.

**TRUE CLASSIFICATION**	**PREDICTED CLASSIFICATION**		
		S. aureus, P. aeruginosa	B. cepacia, S. maltophilia
	S. aureus, P. aeruginosa	23	3
	B. cepacia, S. maltophilia	1	6

**Table 4:** Confusion matrix for CF-patients, classified according to their FEV 1 class.

**TRUE CLASSIFICATION**	**PREDICTED CLASSIFICATION**		
		Mild/Moderate, Severe	Very severe
	Mild/Moderate, Severe	22	3
	Very severe	1	7

## Conclusions

Rheological properties of mucus secretions from 33 Cystic Fibrosis patients were measured both under steady and oscillatory flow regime. The main finding is a strong correlation of the elastic component of the rheological response, as expressed by *G*’, with bacterial colonization and FEV1 values. Such correlation was found to be statistically significant by an advanced statistical analysis based on the PCA method, showing that the types of colonization and FEV1 classes are significantly correlated to the elastic modulus. The latter can be used for CF severity classification with a high predictive efficiency (88%). We therefore propose rheological measurements of mucus elasticity as a new way of CF patient classification, which could be applied as an additional tool for prognostic purposes together with the FEV1 index. Compared to FEV1, rheological tests have the advantage of being less patient dependent, since they are based on measurements of mucus samples with well-established scientific methodology, cheaper and less time consuming. Since a rheometer is not typically available in a clinical laboratory, a further possible application of this work is in the design of a device to measure mucus elasticity. Such a device could be disposable, user-friendly and cheap, and would allow one to use rheological parameters as a prognostic tool in the routine clinical tests. 

Moreover, rheological characterization could allow to monitor the effect of pharmacological treatments (DNase, antibiotic, mucolytic, anti-inflammatory) *in vitro*. Many studies revealed that the high density mucus secretion in CF, and in other pulmonary diseases, has a detrimental effect on drugs delivery, due to their hindered diffusion through the highly viscous mucous layer. The rheological characterization proposed in this work could guide the development of new ways of administering therapeutics with increased local drug concentration in patients with reduced functionality of the CFTR protein [37,38].
